# Supplementary material for: Two Toxoplasma gondii putative pore-forming proteins, GRA47 and GRA72, influence small molecule permeability of the parasitophorous vacuole
Source: mBio. 2024 Feb 21;15(3):e03081-23. doi: 10.1128/mbio.03081-23 (PMC10936148; doi:10.1128/mbio.03081-23)
Supplement: Data S1 — Results color-coded for amino acid conservation performed by PRALINE. [file mbio.03081-23-s0001.pdf]

Results colour-coded for amino acid conservation

The current colourscheme of the alignment is for **amino acid conservation**.

The conservation scoring is performed by PRALINE. The scoring scheme works from 0 for the least conserved alignment position, up to 10 for the most conserved alignment position. The colour assignments are:

Unconserved 0 1 2 3 4 5 6 7 8 9 10 **Conserved**

```
                .........10 .........20 .........30 .........40 .........50
TGCOUG_254000__ MKVVSVHASH PSASGLRGLA SLPRSRAFCV VMARPASRPS RPSTASVGRR
TGPRC2_254000__ MKVVSVHASH PSASGLRGLA SLPRSRAFCV VMARPASRPS RPSTASVGRR
TGARI_254000___ MKVVSVHASH PSASGLRGLA SLPRSRAFCV VMARPASRPS RPSTASVGRR
TGME49_254000__ MKVVSVHASH PSASGLRGLA SLPRSRAFCV VMARPASRPS RPSTASVGRR
TGGT1_254000___ MKVVSVHASH PSASGLRGLA SLPRLRAFCV VMARSASRPS RPSTTSVGRR
TGRH88_004350__ MKVVSVHASH PSASGLRGLA SLPRLRAFCV VMARSASRPS RPSTTSVGRR
TGRUB_254000___ MKVVSVHASH PSASGLRGLA SLPRLRAFCV VMARSASRPS RPSTTSVGRR
TGFOU_254000___ MKVVSVHASH PSASGLRGLA SLPRLRAFCV VMARSASRPS RPSTTSVGRR
TGVAND_254000__ MKVVSVHASH PSASGLRGLA SLPRLRAFCV VMARSASRPS RPSTTSVGRR
TGCAST_254000__ MKVVSVHASH PSASGLRGLA SLPRLRAFCV VMARSASRPS RPSTTSVGRR
TGP89_254000___ MKVVSVHASH PSASGLRGLA SLPRLRAFCV VMARSASRPS RPSTTSVGRR
TGBR9_254000___ MKVVSVHASH PSASGHRGLA SLPRLRAFCV VMARSASRPS RPSTASVGRR
TGMAS_254000___ MKVVSVHASH PSASGHRGLA SLPRLRAFCV VMARSASRPS RPSTASVGRR
TGVEG_254000___ MKVVSVHASH PSASGHRGLA SLPRSRAFCV VMARSASRPS RPSTASVGRR
TGDOM2_254000__ MKVVSVHASH PSASGHRGLA SLPRSRAFCV VMARSASRPS RPSTASVGRR
HHA_254000____H METVSVHASH PSRSGLRGLA SLPRPHVFCV VMARPASRPS LLSAASVGRR
NCLIV_008310___ --METIRPSG SSPSRVAVPV SLPRTCSSSG DVTRSYSRSA LSFA-----R
Ncaninum_LIV_00 --METIRPSG SSPSRVAVPV SLPRTCSSSG DVTRSYSRSA LSFA-----R
BESB_012930____ MVAPARASSL SADAYARRLA GSSSGKRLSL LSFSPAF--- --SRVSTLFA
CSUI_000976____ ---------M PVLFVGRPRG VPRQSRVLPD VTCRAFGVLS GLSYFSSC--
EMWEY_00053180_ ---------- ---------- ---------- --------MK DSLLLPRICF
SN3_02200330___ ---------- ---------- ---------- -MARPDIKDI CNILVSLG--
Consistency     5566766676 7767657667 7777567666 7878878778 6787687657

                .........60 .........70 .........80 .........90 .........100
TGCOUG_254000__ LSGSFLLVAL ALACSPAPPS LSPPSRSP-- ---------- ----------
TGPRC2_254000__ LSGSFLLVAL ALACSPAPPS LSPPSRSP-- ---------- ----------
TGARI_254000___ LSGSFLLVAL ALACSPAPPS LSPPSRSP-- ---------- ----------
TGME49_254000__ LSGSFLLVAL ALACSPAPPS LSPPSRSP-- ---------- ----------
TGGT1_254000___ LAGSFLLVAL ALACSLAPPS LSPPSRSP-- ---------- ----------
TGRH88_004350__ LAGSFLLVAL ALACSLAPPS LSPPSRSP-- ---------- ----------
TGRUB_254000___ LAGSFLLVAL ALACSLAPPS LSPPSRSP-- ---------- ----------
TGFOU_254000___ LAGSFLLVAL ALACSLAPPS LSPPSRSP-- ---------- ----------
TGVAND_254000__ LAGSFLLVAL ALACSLAPPS LSPPSRSP-- ---------- ----------
TGCAST_254000__ LAGSFLLVAL ALACSLAPPS LSPPSRSP-- ---------- ----------
TGP89_254000___ LAGSFLLVAL ALACSLAPPS LSPPSRSP-- ---------- ----------
TGBR9_254000___ LAGSFLLVAL ALACSLAPPS LSPPSRSP-- ---------- ----------
TGMAS_254000___ LAGSFLLVAL ALACSLAPPS LSPPSRSP-- ---------- ----------
TGVEG_254000___ LAGSFLLVAL ALACSLAPPS LSPPSRSP-- ---------- ----------
TGDOM2_254000__ LAGSFLLVAL ALACSLAPPS LSPPSRSP-- ---------- ----------
HHA_254000____H IAGSFLLVAL ALACSPAPPS LSPPSQST-- ---------- ----------
NCLIV_008310___ DLGASCVFAL VLFCALSS-S LSSDRLSP-- ---------- ----------
Ncaninum_LIV_00 DLGASCVFAL VLFCALSS-S LSSDRLSP-- ---------- ----------
BESB_012930____ VPASLCVLFL ASSCFSLPPT STPTSSPAHA SHLSLSTSSP SDRRAHASSS
CSUI_000976____ VFLQSLFLFL WYSCCESIFC LFSPAEAA-- ---------- ----------
EMWEY_00053180_ LFVLFFFIFL NIHKKEVSAA DSASSVTL-- ---------- ----------
SN3_02200330___ ----FVGGAL GLYVLLAHGA YGS------- ---------- ----------
Consistency     767887888* 8878869879 8997868700 0000000000 0000000000

                .........110.........120.........130.........140.........150
TGCOUG_254000__ ---------T SFLPNASFA- --------AC MAPPPPNQIG DIKRVVLRLE
TGPRC2_254000__ ---------T SFLPNASFA- --------AC MAPPPPNQIG DIKRVVLRLE
TGARI_254000___ ---------T SFLPNASFA- --------AC MAPPPPNQIG DIKRVVLRLE
TGME49_254000__ ---------T SFLPNASFA- --------AC MAPPPPNQIG DIKRVVLRLE
TGGT1_254000___ ---------T SFLPNASFA- --------AC MAPPPPNQIG DIKRVVLRLE
TGRH88_004350__ ---------T SFLPNASFA- --------AC MAPPPPNQIG DIKRVVLRLE
TGRUB_254000___ ---------T SFLPNASFA- --------AC MAPPPPNQIG DIKRVVLRLE
TGFOU_254000___ ---------T SFLPNASFA- --------AC MAPPPPNQIG DIKRVVLRLE
TGVAND_254000__ ---------T SFLPNASFA- --------AC MAPPPPNQIG DIKRVVLRLE
TGCAST_254000__ ---------T SFLPNASFA- --------AC MAPPPPNQIG DIKRVVLRLE
TGP89_254000___ ---------T SFLPNASFA- --------AC MAPPPPNQIG DIKRVVLRLE
TGBR9_254000___ ---------T SFLPNASFA- --------AC MAPPPPNQIG DIKRVVLRLE
TGMAS_254000___ ---------T SFLPNASFA- --------AC MAPPPPNQIG DIKRVVLRLE
TGVEG_254000___ ---------T SFLPNASFA- --------AC MAPPPPNQIG DIKRVVLRLE
```

```
TGDOM2_254000__ ---------T SFLPNASFA- --------AC MAPPPPNQIG DIKRVVLRLE
HHA_254000____H ---------A SFLPSAGFT- --------AC MAPPPPNQFG DINRVVLRLE
NCLIV_008310___ ---------S SLPRASFAA- --------GV GAPPTGEQFR DIKRVLLRLE
Ncaninum_LIV_00 ---------S SLPRASFAA- --------GV GAPPTGEQFR DIKRVLLRLE
BESB_012930____ SSGANTHSYA SSVPHFSFAE GSDTALGVSP LSPGGGHLPG NIKSVMLRVE
CSUI_000976____ ---------- ---------- ---------- -SDSASLSGG EVRHVRLFIE
EMWEY_00053180_ ---------- ---------- ---------- EDRVAADTPW GVRSGATTPT
SN3_02200330___ ---------- ---------- ---------- VTQPEAGESR SVSDLALTVQ
Consistency     0000000007 7666666670 0000000076 7988888877 9998989889

                .........160.........170.........180.........190.........200
TGCOUG_254000__ TKDGKVYDLN VGGLQAVAGG APQLGVTP-- --------GV GVSGSGAARP
TGPRC2_254000__ TKDGKVYDLN VGGLQAVAGG APQLGVTP-- --------GV GVSGSGAARP
TGARI_254000___ TKDGKVYDLN VGGLQAVAGG APQLGVTP-- --------GV GVSGSGAARP
TGME49_254000__ TKDGKVYDLN VGGLQAVAGG APQLGVTP-- --------GV GVSGSGAARP
TGGT1_254000___ AKDGKVYDLN VGGLQAVAGG APQLGVTP-- --------GV GVSGSGAARP
TGRH88_004350__ AKDGKVYDLN VGGLQAVAGG APQLGVTP-- --------GV GVSGSGAARP
TGRUB_254000___ AKDGKVYDLN VGGLQAVAGG APQLGVTP-- --------GV GVSGSGAARP
TGFOU_254000___ AKDGKVYDLN VGGLQAVAGG APQLGVTP-- --------GV GVSGSGAARP
TGVAND_254000__ AKDGKVYDLN VGGLQAVAGG APQLGVTP-- --------GV GVSGSGAARP
TGCAST_254000__ AKDGKVYDLN VGGLQAVAGG APQLGVTP-- --------GV GVSGSGAARP
TGP89_254000___ AKDGKVYDLN VGGLQAVAGG APQLGVTP-- --------GV GVSGSGAARP
TGBR9_254000___ AKDGKVYDLN VGGLQAVAGG APQLGVTP-- --------GV GVSGSGAARP
TGMAS_254000___ AKDGKVYDLN VGGLQAVAGG APQLGVTP-- --------GV GVSGSGAARP
TGVEG_254000___ AKDGKVYDLN VGGLQAVAGG APQLGVTP-- --------GV GVSGSGAARP
TGDOM2_254000__ AKDGKVYDLN VGGLQAVAGG APQLGVTP-- --------GV GVSGSGAARP
HHA_254000____H AKDGKVYDLN VGGLHAVAGG APQSGLTPGF VTPGSGASGV EASGSGAPGP
NCLIV_008310___ AHDGRVYDLD VGGLHAVAGG QPMGAAPG-- --------HL APGAAGHQRE
Ncaninum_LIV_00 AHDGRVYDLD VGGLHAVAGG QPMGAAPG-- --------HL APGAAGHQRE
BESB_012930____ GEDGRFYNLD VASVYAVAPT PGSQGAPVLY ------PASA GAAALPPATA
CSUI_000976____ TKDGRQFDLY NADNVQIQPA SGSRDR---- ---------L LIAPGGPGTS
EMWEY_00053180_ TKPADDVYLT KREHNNDNGP SIKLKV---- ---------V DGNGRVAEIP
SN3_02200330___ MADGRTYKYH ---------- ---------- ---------- ---PSPSVRA
Consistency     8899989898 8887788888 8776876600 0000000068 7788888888

                .........210.........220.........230.........240.........250
TGCOUG_254000__ VD-RSAQERR PSMWSRVSQH AANTLVASVL FLSIGEAYSW LKRHSQEKQK
TGPRC2_254000__ VD-RSAQERR PSMWSRVSQH AANTLVASVL FLSIGEAYSW LKRHSQEKQK
TGARI_254000___ VD-RSAQERR PSMWSRVSQH AANTLVASVL FLSIGEAYSW LKRHSQEKQK
TGME49_254000__ VD-RSAQERR PGMWSRVSQH AANTLVASVL FLSIGEAYSW LKRHSQEKQK
TGGT1_254000___ VD-RSAQERR PSMWSRVSQH AANTLVASVL FLSIGEAYSW LKRHSQEKQK
TGRH88_004350__ VD-RSAQERR PSMWSRVSQH AANTLVASVL FLSIGEAYSW LKRHSQEKQK
TGRUB_254000___ VD-RSAQERR PSMWSRVSQH AANTLVASVL FLSIGEAYSW LKRHSQEKQK
TGFOU_254000___ VD-RSAQERR PSMWSRVSQH AANTLVASVL FLSIGEAYSW LKRHSQEKQK
TGVAND_254000__ VD-RSAQERR PSMWSRVSQH AANTLVASVL FLSIGEAYSW LKRHSQEKQK
TGCAST_254000__ VD-RSAQERR PSMWSRVSQH AANTLVASVL FLSIGEAYSW LKRHSQEKQK
TGP89_254000___ VD-RSAQERR PSMWSRVSQH AANTLVASVL FLSIGEAYSW LKRHSQEKQK
TGBR9_254000___ VD-RSAQERR PSMWSRVSQH AANTLVASVL FLSIGEAYSW LKRHSQEKQK
TGMAS_254000___ VD-RSAQERR PSMWSRVSQH AANTLVASVL FLSIGEAYSW LKRHSQEKQK
TGVEG_254000___ VD-RSAQERR PSMWSRVSQH AANTLVASVL FLSIGELYSW LKRHSQEKQK
TGDOM2_254000__ VD-RSAQERR PSMWSRVSQH AANTLVASVL FLSIGELYSW LKRHSQEKQK
HHA_254000____H VV-PPPQEQR PSMWSRVSQH AANTLVASIL FLSIGEAYSW LKRRSQEKQK
NCLIV_008310___ LP-AGAPEQK PSTWRKISHH AANTVVASVL FLAIGEAYNW LKRRRQQQRM
Ncaninum_LIV_00 LP-AGAPEQK PSTWRKISHH AANTVVASVL FLAIGEAYNW LKRRRQQQRM
BESB_012930____ AG-VQAPEEK SSMWKMVGQH AVSTAVASFL LLAMGEAYSW FKRRSQEKKM
CSUI_000976____ GG-TVSPPQE KSMWRSVAHH AVSTVVASAL LLGIGEVYSW WKSRRQEKRM
EMWEY_00053180_ IDFHEARIQP RGFWGDVGVN GMALFLASAC LLLIKEIFSF ISSRRERKLN
SN3_02200330___ VPLAPAPAPE GWFRSFVRNF SVGITVPLVL -LGIGELLSW MKRRHSSRAM
Consistency     8807898888 8989889999 9999899999 8*899*8999 8998899988

                .........260.........270.........280.........290.........300
TGCOUG_254000__ RLAREEVARE KAAIASRLRE VEERLTVLED VEEAAERGEG --QLTGKQKA
TGPRC2_254000__ RLAREEVARE KAAIASRLRE VEERLTVLED VEEAAERGEG --QLTGKQKA
TGARI_254000___ RLAREEVARE KAAIASRLRE VEERLTVLED VEEAAERGEG --QLTGKQKA
TGME49_254000__ RLAREEVARE KAAIASRLRE VEERLTVLED VEEAAERGEG --QLTGKQKA
TGGT1_254000___ RLAREEVARE KAAIESRLRE VEERLTVLED VEEAAERGEG --QLTGKQKA
TGRH88_004350__ RLAREEVARE KAAIESRLRE VEERLTVLED VEEAAERGEG --QLTGKQKA
TGRUB_254000___ RLAREEVARE KAAIESRLRE VEERLTVLED VEEAAERGEG --QLTGKQKA
TGFOU_254000___ RLAREEVARE KAAIESRLRE VEERLTVLED VEEAAERGEG --QLTGKQKA
TGVAND_254000__ RLAREEVARE KAAIESRLRE VEERLTVLED VEEAAERGEG --QLTGKQKA
TGCAST_254000__ RLAREEVARE KAAIESRLRE VEERLTVLED VEEAAERGEG --QLTGKQKA
TGP89_254000___ RLAREEVARE KAAIESRLRE VEERLTVLED VEEAAERGEG --QLTGKQKA
TGBR9_254000___ RLAREEVARE KAAIESRLRE VEERLTVLED VEEAAERGEG --QLTGKQKA
TGMAS_254000___ RLAREEVARE KAAIESRLRE VEERLTVLED VEEAAERGEG --QLTGKQKA
TGVEG_254000___ RLAREEVARE KAAIESRLRE VEERLTVLED VEEAAERGEG --QLTGKQKA
```

```
TGDOM2_254000__ RLAREEVARE KAAIESRLRE VEERLTVLED VEEAAERGEG --QLTGKQKA
HHA_254000____H RLAREDVARE KAAIASRLRE VEERLTALED VEEAAERGEG --QLTGKQKA
NCLIV_008310___ QQVREELERE KEMLALQLKS VEQRLEGLEQ LEAEEERGEP GKRMTGKQKA
Ncaninum_LIV_00 QQVREELERE KEMLALQLKS VEQRLEGLEQ LEAEEERGEP GKRMTGKQKA
BESB_012930____ NKWRQELAQE REMLVAELRA VERKLRALEK LEVASEEEDK --KMTARQRA
CSUI_000976____ AHLRKEVANE REQVAREMRR VKRRLAVFTG LGD------- --RLTTSQRA
EMWEY_00053180_ IAQRQNAAQR RAFITSRLEE ADQELQEIEE NIQEN----- ----IQNKEE
SN3_02200330___ KDKARRRVQE RRRILRDMYA AQEELQALEE KLEAKRMDAG --TNEQDSRA
Consistency     877999899 9879788988 9989*88999 8888877777 0078989999

                .........310.........320.........330.........340.........350
TGCOUG_254000__ DLSILRSERE DLLAQQRRNA LRDGQLNGFP V-KKTSFASL LDGRSRSRVR
TGPRC2_254000__ DLSILRSERE DLLAQQRRNA LRDGQLNGFP V-KKTSFASL LDGRSRSRVR
TGARI_254000___ DLSILRSERE DLLAQQRRNA LRDGQLNGFP V-KKTSFASL LDGRSRSRVR
TGME49_254000__ DLSILRSERE DLLAQQRRNA LRDGQLNGFP V-KKTSFASL LDGRSRSRVR
TGGT1_254000___ DLSILRSERE DLLAQQRRNA LRDGQLNGFP V-KKTSFASL LDGRSRSRVR
TGRH88_004350__ DLSILRSERE DLLAQQRRNA LRDGQLNGFP V-KKTSFASL LDGRSRSRVR
TGRUB_254000___ DLSILRSERE DLLAQQRRNA LRDGQLNGFP V-KKTSFASL LDGRSRSRVR
TGFOU_254000___ DLSILRSERE DLLAQQRRNA LRDGQLNGFP V-KKTSFASL LDGRSRSRVR
TGVAND_254000__ DLSILRSERE DLLAQQRRNA LRDGQLNGFP V-KKTSFASL LDGRSRSRVR
TGCAST_254000__ DLSILRSERE DLLAQQRRNA LRDGQLNGFP V-KKTSFASL LDGRSRSRVR
TGP89_254000___ DLSILRSERE DLLAQQRRNA LRDGQLNGFP V-KKTSFASL LDGRSRSRVR
TGBR9_254000___ DLSILRSERE DLLAQQRRNA LRDGQLNGFP V-KKTSFASL LDGRSRSRVR
TGMAS_254000___ DLSILRSERE DLLAQQRRNA LRDGQLNGFP V-KKTSFASL LDGRSRSRVR
TGVEG_254000___ DLSILRSERE DLLAQQRRNA LRDGQLNGFP V-KKTSFASL LDGRSRSRVR
TGDOM2_254000__ DLSILRSERE DLLAQQRRNA LRDGQLNGFP V-KKTSFASL LDGRSRSRVR
HHA_254000____H DLSILRSERE DLLAQQRRNA FRDGQLNGFP V-KKTSFASL LDGRSRSSGR
NCLIV_008310___ ELSMLRKERE ELVIQQQRNE LRDGQLNGFP VAKKATFSGV AGFGNKAKKS
Ncaninum_LIV_00 ELSMLRKERE ELVIQQQRNE LRDGQLNGFP VAKKATFSGV AGFGNKAKKS
BESB_012930____ QLASLREEKE DLTYQLRRNE FQTNQVYGFP GGKPGAAGGV AGVAARA--S
CSUI_000976____ EKLNLQDQLE DLKYQLRRNE LKASQLYGFP L-SSKPYQRT AAGNA-----
EMWEY_00053180_ NIKPLRKILQ SL--DLYREE LRDADAPAV- ----LAYLDH ILQRTKMLIR
SN3_02200330___ LNASYKARKQ ELLFRLEDLR YQLRDMYEFA TTGSADAGEF EGAADYDDQR
Consistency     8898998889 9*77988998 8988998998 8088898888 7878988668

                .........360.........370.........380.........390.........400
TGCOUG_254000__ RGSKESRRQN SGSQSDEEDV EESDEDARAL RADDRDGRSA PGAAKNFLDR
TGPRC2_254000__ RGSKESRRQN SGSQSDEEDV EESDEDARAL RADDRDGRSA PGAAKNFLDR
TGARI_254000___ RGSKESRRQN SGSQSDEEDV EESDEDARAL RADDRDGRSA PGAAKNFLDR
TGME49_254000__ RGSKESRRQN SGSQSDEEDV EESDEDARAL RADDRDGRSA PGAAKNFLDR
TGGT1_254000___ RGSKESRRQN SGSQSDEEDV EESDEDARAL RADDRDGRSA PGAAKNFLDR
TGRH88_004350__ RGSKESRRQN SGSQSDEEDV EESDEDARAL RADDRDGRSA PGAAKNFLDR
TGRUB_254000___ RGSKESRRQN SGSQSDEEDV EESDEDARAL RADDRDGRSA PGAAKNFLDR
TGFOU_254000___ RGSKESRRQN SGSQSDEEDV EESDEDARAL RADDRDGRSA PGAAKNFLDR
TGVAND_254000__ RGSKESRRQN SGSQSDEEDV EESDEDARAL RADDRDGRSA PGAAKNFLDR
TGCAST_254000__ RGSKESRRQN SGSQSDEEDV EESDEDARAL RADDRDGRSA PGAAKNFLDR
TGP89_254000___ RGSKESRRQN SGSQSDEEDV EESDEDARAL RADDRDGRSA PGAAKNFLDR
TGBR9_254000___ RGSKESRRQN SGSQSDEEDV EESDEDARAL RADDRDGRSA PGAAKNFLDR
TGMAS_254000___ RGSKESRRQN SGSQSDEEDV EESDEDARAL RADDRDGRSA PGAAKNFLDR
TGVEG_254000___ RGSKESRRQN SGSQSDEEDV EESDEDARAL RADDRDGRSA PGAAKNFLDR
TGDOM2_254000__ RGSKESRRQN SGSQSDEEDV EESDEDARAL RADDRDGRSA PGAAKNFLDR
HHA_254000____H GASRESRRRD SGSQSDEEDV EESDEDIRTL RTEDRDGRSA PGAAKNTLDQ
NCLIV_008310___ RGNKNGSRKR DNRSGDEDVT EESDPDFRPP RDGRGNEDSS PDALDSVTSP
Ncaninum_LIV_00 RGNKNGSRKR DNRSGDEDVT EESDPDFRPP RDGRGNEDSS PDALDSVTSP
BESB_012930____ RAFRGTRGKG VVTSSRSQRS ESEDETVAEE EEETEEEDDF PPSEGGVTGH
CSUI_000976____ -GGQRSSLRY GEALEQDEED ERSTESEDTF RGTTKKRTIF DGGQ------
EMWEY_00053180_ KSSSSSSSNN SSKGTFAHAD DLDDDDLLQI NVSSSSSHAA ATAA------
SN3_02200330___ SSRRPSRAPD RRNHSANYAA DDRHASRTAD FVHAAQGRSS TNS-------
Consistency     8989898888 8888989888 9899897887 8888798898 9897675666

                .........410.........420.........430.........440.........450
TGCOUG_254000__ R--------- ---------- --------QI ERSGAKKGDA GSEETQKALA
TGPRC2_254000__ R--------- ---------- --------QI ERSGAKKGDE GSEETQKALA
TGARI_254000___ R--------- ---------- --------QI ERSGAKKGDE GSEETQKALA
TGME49_254000__ R--------- ---------- --------QI ERSGAKKGDE GSEETQKALA
TGGT1_254000___ R--------- ---------- --------QI ERSGAKKGDE GSEETQKALA
TGRH88_004350__ R--------- ---------- --------QI ERSGAKKGDE GSEETQKALA
TGRUB_254000___ R--------- ---------- --------QI ERSGAKKGDE GSEETQKALA
TGFOU_254000___ R--------- ---------- --------QI ERSGAKKGDE GSEETQKALA
TGVAND_254000__ R--------- ---------- --------QI ERSGAKKGDE GSEETQKALA
TGCAST_254000__ R--------- ---------- --------QI ERSGAKKGDE GSEETQKALA
TGP89_254000___ R--------- ---------- --------QI ERSGAKKGDE GSEETQKALA
TGBR9_254000___ R--------- ---------- --------QI ERSGAKKGDE GSEETQKALA
TGMAS_254000___ R--------- ---------- --------QI ERSGAKKGDE GSEETQKALA
TGVEG_254000___ R--------- ---------- --------QI ERSGAKKGDE GSEETQKALA
```

```
TGDOM2_254000__    R--------- ---------- --------QI ERSGAKKGDE GSEETQKALA
HHA_254000____H    M--------- ---------- --------QI EGLGTKKGAA GSEETQKTPA
NCLIV_008310___    RGANEEAQSS LLRRGEHVDT PERWGTGVQS TAASAKRGQT PEQGRAAGIG
Ncaninum_LIV_00    RGANEEAQSS LLRRGEHVDT PERWGTGVQS TAASAKRGQT PEQGRAAGIG
BESB_012930____    R--------- ---------- ---------- LGDGGTDSAA RAGDKDGAIS
CSUI_000976____    ---------- ---------- ---------- ---AEEKNKA TAADSTGDVA
EMWEY_00053180_    ---------- ---------- ---------- --------TA AAAATTKAAE
SN3_02200330___    ---------- ---------- ---------- ---------- ---------T
Consistency        7000000000 0000000000 0000000066 6567777777 7888877879

                   .........460.........470.........480.........490.........500
TGCOUG_254000__    GDSEEKASKK -TGETK---T AEAGDAGNAG ---NAGEVGR P---------
TGPRC2_254000__    GDSEEKASKK -TGETK---T AEAGDAGNAG ---NAGEVGR P---------
TGARI_254000___    GDSEEKASKK -TGETK---T AEAGDAGNAG ---NAGEVGR P---------
TGME49_254000__    GDSEEKASKK -TGETK---T AEAGDAGNAG ---NAGEVGR P---------
TGGT1_254000___    GDSEEKASKK -TGETKTAEA GDAGNAGNAG ---KAGEVGR P---------
TGRH88_004350__    GDSEEKASKK -TGETKTAEA GDAGNAGNAG ---KAGEVGR P---------
TGRUB_254000___    GDSEEKASKK -TGETKTAEA GDAGNAGNAG ---KAGEVGR P---------
TGFOU_254000___    GDSEEKASKK -TGETKTAEA GDAGNAGNAG ---KAGEVGR P---------
TGVAND_254000__    GDSEEKASKK -TGETKTAEA GDAGNAGNAG ---KAGEVGR P---------
TGCAST_254000__    GDSEEKASKK -TGETKTAEA GDAGNAGNAG ---KAGEVGR P---------
TGP89_254000___    GDSEEKASKK -TGETKTAEA GDAGNAGNAG ---KAGEVGR P---------
TGBR9_254000___    GDSEEKASKK -TGETKTAEA GDAGNAGNAG ---NAGEVGR P---------
TGMAS_254000___    GDSEEKASKK -TGETKTAEA GDAGNAGNAG ---NAGEVGR P---------
TGVEG_254000___    GDSEEKASKK -TGET----T AEAGDAGNAG ---NAGEVGR P---------
TGDOM2_254000__    GDSEEKASKK -TGET----T AEAGDAGNAG ---NAGEVGR P---------
HHA_254000____H    GDSEEKGSKR -ASETKTAGA GDAGNADEVG NARNAGEAER T---------
NCLIV_008310___    SKETETASTP LATGSGNVSA EESAKDMNAG EAAEAKAIGR RNEGEKGPVA
Ncaninum_LIV_00    SKETETASTP LATGSGNVSA EESAKDMNAG EAAEAKAIGR RNEGEKGPVA
BESB_012930____    GAEQAGANSD AGATETGAAA AKREKSGRE- ---KDTREAK T---------
CSUI_000976____    EDNEQEETQR RTETAQRTET SARVAG---- ---------- ----------
EMWEY_00053180_    STASSTTKQE MINDSISSDS STTTN----- ---------- ----------
SN3_02200330___    GRSEEPAQRR ---------- ---------- ---------- ----------
Consistency        9899989988 0878853438 7887876766 0006766667 6000000000

                   .........510.........520.........530.........540.........550
TGCOUG_254000__    ---------- -------RSQ TRERKSQRKD AKERSHASED EDDFALLRSG
TGPRC2_254000__    ---------- -------RSQ TRERKSQRKD AKERSHASED EDDFALLRSG
TGARI_254000___    ---------- -------RSQ TRERKSQRKD AKERSHASED EDDFALLRSG
TGME49_254000__    ---------- -------RSQ TRERKSQRKD AKERSHASED EDDFALLRSG
TGGT1_254000___    ---------- -------RSQ TRERKSQRKD AKERSHASED EDDFALLRSG
TGRH88_004350__    ---------- -------RSQ TRERKSQRKD AKERSHASED EDDFALLRSG
TGRUB_254000___    ---------- -------RSQ TRERKSQRKD AKERSHASED EDDFALLRSG
TGFOU_254000___    ---------- -------RSQ TRERKSQRKD AKERSHASED EDDFALLRSG
TGVAND_254000__    ---------- -------RSQ TRERKSQRKD AKERSHASED EDDFALLRSG
TGCAST_254000__    ---------- -------RSQ TRERKSQRKD AKERSHASED EDDFALLRSG
TGP89_254000___    ---------- -------RSQ TRERKSQRKD AKERSHASED EDDFALLRSG
TGBR9_254000___    ---------- -------RSQ TRERKSQRKD AKERSHASED EDDFALLRSG
TGMAS_254000___    ---------- -------RSQ TRERKSQRKD AKERSHASED EDDFALLRSG
TGVEG_254000___    ---------- -------RSQ TRERKSQRKD AKERSHASED EDDFALLRSG
TGDOM2_254000__    ---------- -------RSQ TRERKSQRKD AKERSHASED EDDFALLRSG
HHA_254000____H    ---------- -------KIQ TRERKSQRKD AKERSRAFED EDDFSLLRRG
NCLIV_008310___    GETVETGFRD KRRGNEGKKA THERVAGREP SEKKIQVAED EDDEGLMQGE
Ncaninum_LIV_00    GETVETGFRD KRRGNEGKKA THERVAGREP SEKKIQVAED EDDEGLMQGE
BESB_012930____    ---------- -------ADA TAGPRRKKNG DARRPKSALE EEDEALVRGD
CSUI_000976____    ---------- -------KSI PRGSQSYNKG RSPPQSSGTR QEQFTRRRA-
EMWEY_00053180_    ---------- ---------Q QQQQQQQQQQ QQQQEQQQQQ QQQQQKLKPD
SN3_02200330___    ---------- -------LQE DRQFRDHTRQ GSESKRPGRQ N------RSS
Consistency        0000000000 0000000778 9898898888 8888888889 9886888987

                   .........560.........570.........580.........590.........600
TGCOUG_254000__    GSSGSEKMKY ITQLLAEHPG MSPEEAIEML NFMSNGKRGS ----------
TGPRC2_254000__    GSSGSEKMKY ITQLLAEHPG MSPEEAIEML NFMSNGKRGS ----------
TGARI_254000___    GSSGSEKMKY ITQLLAEHPG MSPEEAIEML NFMSNGKRGS ----------
TGME49_254000__    GSSGSEKMKY ITQLLAEHPG MSPEEAIEML NFMSNGKRGS ----------
TGGT1_254000___    GSSGSEKMKY ITQLLAEHPG MSPEEAIEML NFMSNGKRGS ----------
TGRH88_004350__    GSSGSEKMKY ITQLLAEHPG MSPEEAIEML NFMSNGKRGS ----------
TGRUB_254000___    GSSGSEKMKY ITQLLAEHPG MSPEEAIEML NFMSNGKRGS ----------
TGFOU_254000___    GSSGSEKMKY ITQLLAEHPG MSPEEAIEML NFMSNGKRGS ----------
TGVAND_254000__    GSSGSEKMKY ITQLLAEHPG MSPEEAIEML NFMSNGKRGS ----------
TGCAST_254000__    GSSGSEKMKY ITQLLAEHPG MSPEEAIEML NFMSNGKRGS ----------
TGP89_254000___    GSSGSEKMKY ITQLLAEHPG MSPEEAIEML NFMSNGKRGS ----------
TGBR9_254000___    GSSGSEKMKY ITQLLAEHPG MSPEEAIEML NFMSNGKRGS ----------
TGMAS_254000___    GSSGSEKMKY ITQLLAEHPG MSPEEAIEML NFMSNGKRGS ----------
TGVEG_254000___    GSSGSEKMKY ITQLLAEHPG MSPEEAIEML NFMSNGKRGS ----------
```

```
TGDOM2_254000__GSSGSEKMKY ITQLLAEHPG MSPEEAIEML NFMSNGKRGS ----------
HHA_254000____HGSSGSEKMKY IAQLLAEHPG MSPEEAIGML NFLSNSKRGT ----------
NCLIV_008310___ASLGAKKLRY ISQLLQEHPG LSPEEAIDMV HFMSKGHPHE NHAKRNRGTT
Ncaninum_LIV_00ASLGAKKLRY ISQLLQEHPG LSPEEAIDMV HFMSKGHPHE NHAKRNRGTT
BESB_012930____GGLPAERMRY MTQILQEHPG LSPAEAIQMA NQIQASEQAR AHRRWP----
CSUI_000976____---SADQLRY IKQLLQEYPG VTPDEAIKMA AQVPLSGQQR ASSRQ-----
EMWEY_00053180_-------IEM VQMLAQQNPH LTPPEVIRLA QELMQKRGAN IKKNKYN---
SN3_02200330___KRAGQSRVEV IRQLLDEVPG MPPEEVLRIA E--KAAPRGL ----------
Consistency    7768888988 98999898*9 99*9*99898 9788888888 0000000000

               ...
TGCOUG_254000__---
TGPRC2_254000__---
TGARI_254000___---
TGME49_254000__---
TGGT1_254000___---
TGRH88_004350__---
TGRUB_254000___---
TGFOU_254000___---
TGVAND_254000__---
TGCAST_254000__---
TGP89_254000___---
TGBR9_254000___---
TGMAS_254000___---
TGVEG_254000___---
TGDOM2_254000__---
HHA_254000____H---
NCLIV_008310___GGP
Ncaninum_LIV_00GGP
BESB_012930____---
CSUI_000976____---
EMWEY_00053180_---
SN3_02200330___---
Consistency    000
```
