# Supplementary material for: Two Toxoplasma gondii putative pore-forming proteins, GRA47 and GRA72, influence small molecule permeability of the parasitophorous vacuole
Source: mBio. 2024 Feb 21;15(3):e03081-23. doi: 10.1128/mbio.03081-23 (PMC10936148; doi:10.1128/mbio.03081-23)
Supplement: Supplemental material — Figures S1 to S8 and captions for supplemental data. [file mbio.03081-23-s0002.docx]

**Supplementary Table 1A**

List of primer sequences, and oligonucleotides utilized for sgRNA constructs

**Supplementary Table 1B**

Details regarding the antibodies employed in the study.

**Supplementary Table 1C**

Presents the total counts of unique peptides and spectra obtained from the immunoprecipitations involving GRA72 and GRA57.

**Supplementary Table 1D**

Contains information about the constructs of GRA17, GRA15, GRA47 and GRA72 used for *Xenopus* experiments.

**Supplementary data 1**

Praline-generated alignment of GRA47 orthologues. The results are color-coded for amino acid conservation of GRA47.


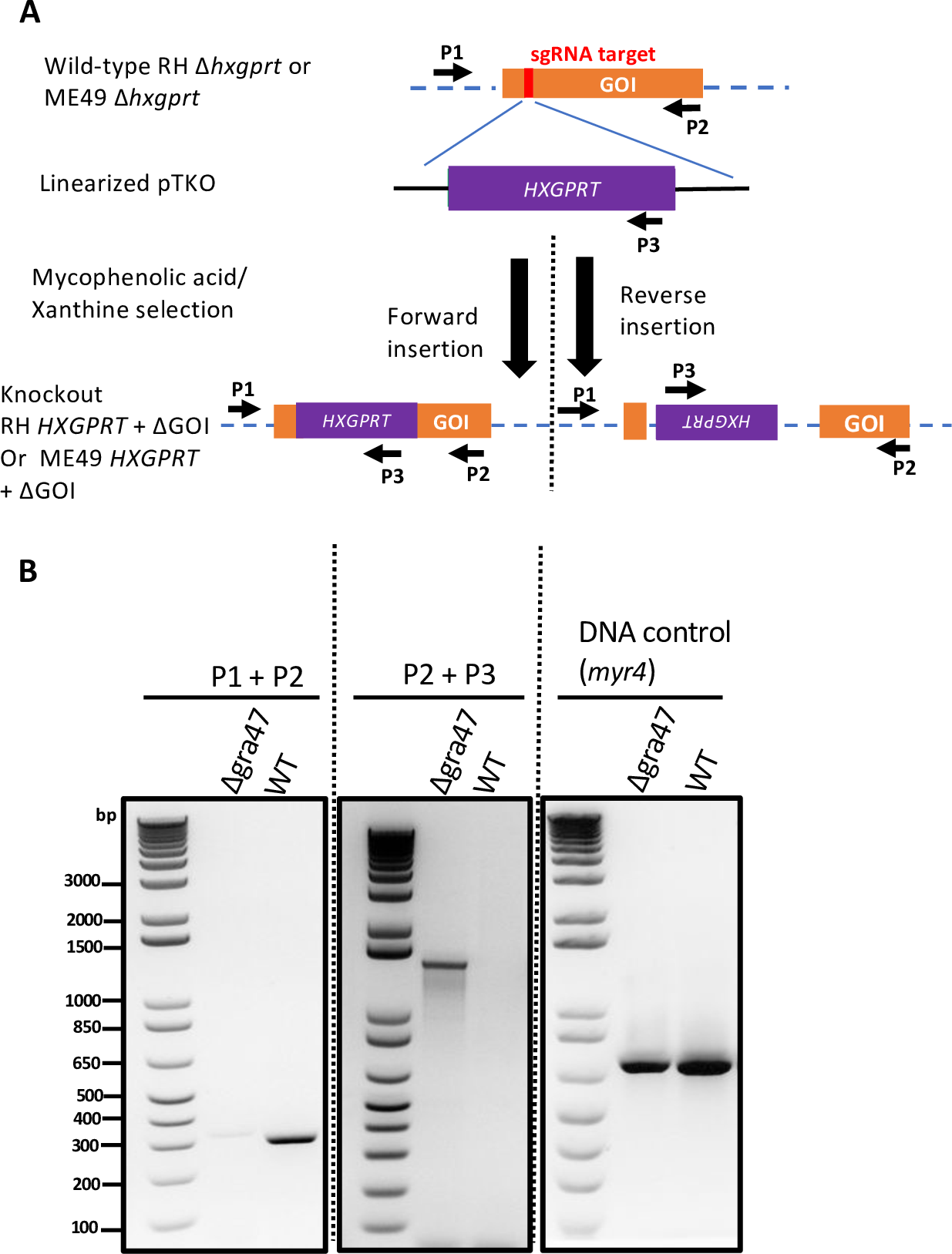


**FIG S1** Generation of knockout parasite strains **A)** The diagram illustrates the strategy employed to delete *GRA47* in both the type RH and ME49 strains. The CRISPR/Cas9-targeting site, depicted within a red box, indicates the specific region targeted for modification. To accomplish this, a linearized pTKO plasmid containing an *HXGPRT* selection cassette was utilized as a repair template. The selection process was conducted using mycophenolic acid and xanthine. **B)** Confirmation of the disruption of the gene of interest (GOI) was achieved using primers P1 and P2, which amplified a region within the GOI of 320 base pairs. For quality control of the PCR, amplification of MYR4 was used. To verify the successful insertion of the repair template, primers P2 and P3 were employed.

**
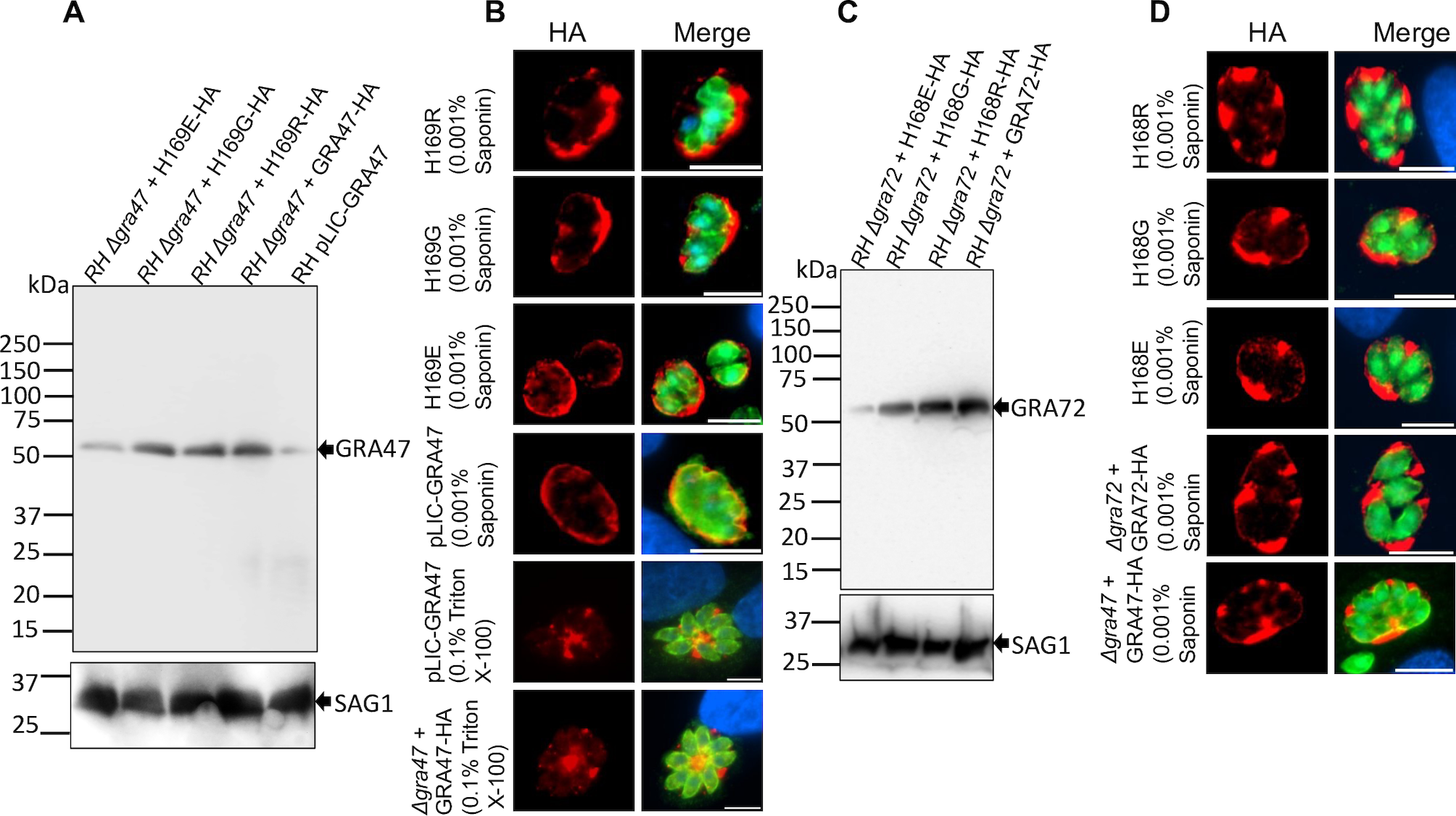
**

**FIG S2** Complementation of Δ*gra47* and Δ*gra72* parasites with wild-type or histidine mutant derivatives. **A and C)** Shown is a Western blot demonstrating the successful complementation of the Δ*gra47* or Δ*gra72* knockout parasites in the type 1 (RH) background or C-terminally HA endotagged GRA47. SAG1 was used as the parasite loading control. Predicted molecular weights: GRA47= 51.54 kDa, SAG1= 34.83 kDa, GRA72= 57.51kDa.

**B)** An immunofluorescence Assay (IFA) showing the localization of GRA47 from C-terminally HA-tagged or complemented Δ*gra47* parasites, marked in red, using different permeabilization methods. The scale bar represents 8μM. **D)** IFA showing the localization of Δ*gra72* parasites complemented with wild-type GRA72 or GRA72 containing histidine mutants. The scale bar used was 8μM.


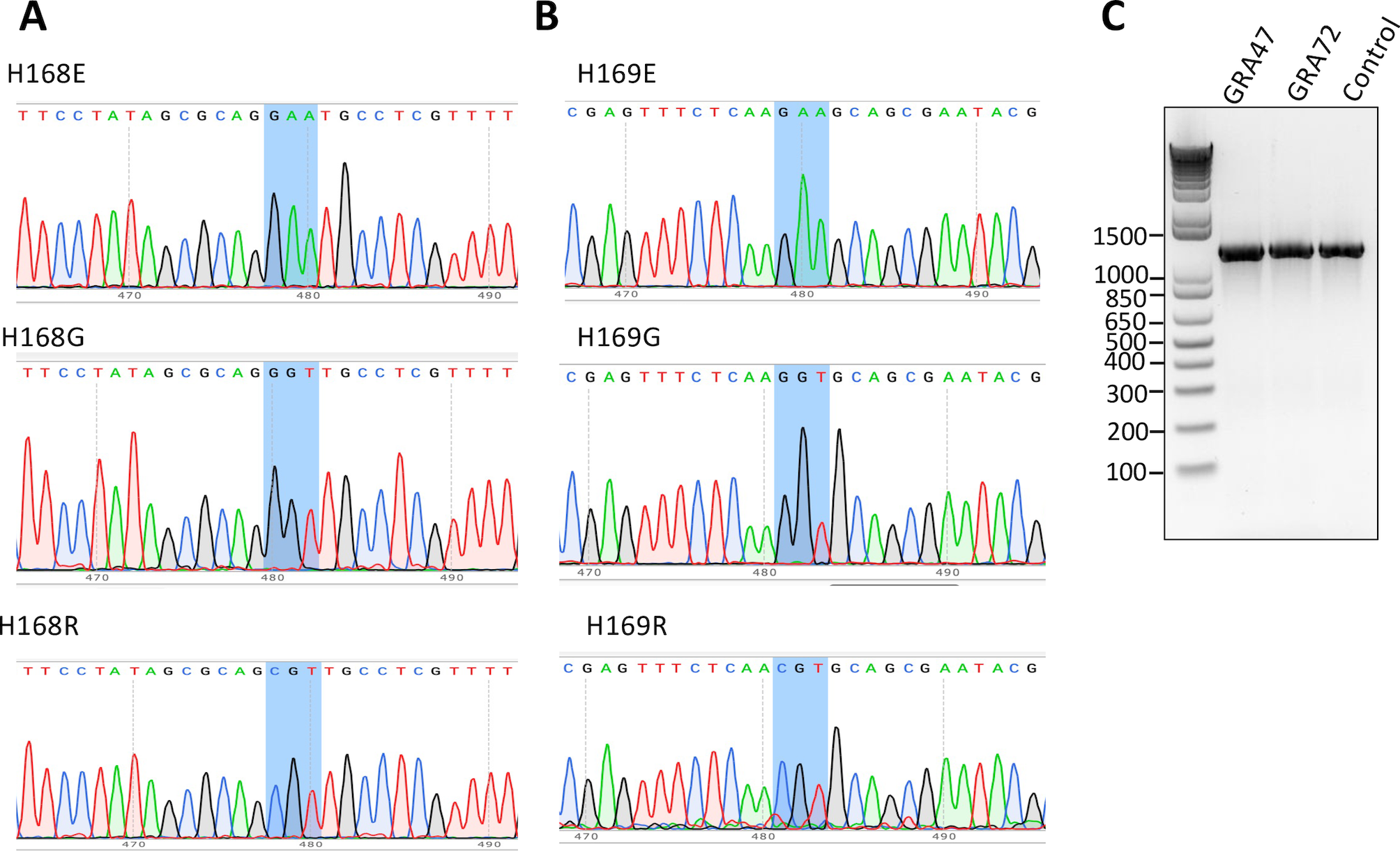


**FIG S3** Confirmation of histidine mutations. Genomic DNA was extracted from **A)** GRA72 and **B)** GRA47 histidine mutant parasites and PCR amplification was performed. The PCR amplicon was Sanger sequenced. Shown are histidine mutations into glutamic acid (H168E and H169E), glycine (H168G and H169G) and arginine (H168R and H169R) of GRA72 and GRA47, respectively. **C)** Agarose gel electrophoresis showing the correct size of GRA47 and GRA72. Control is wild-type DNA without mutation.

**
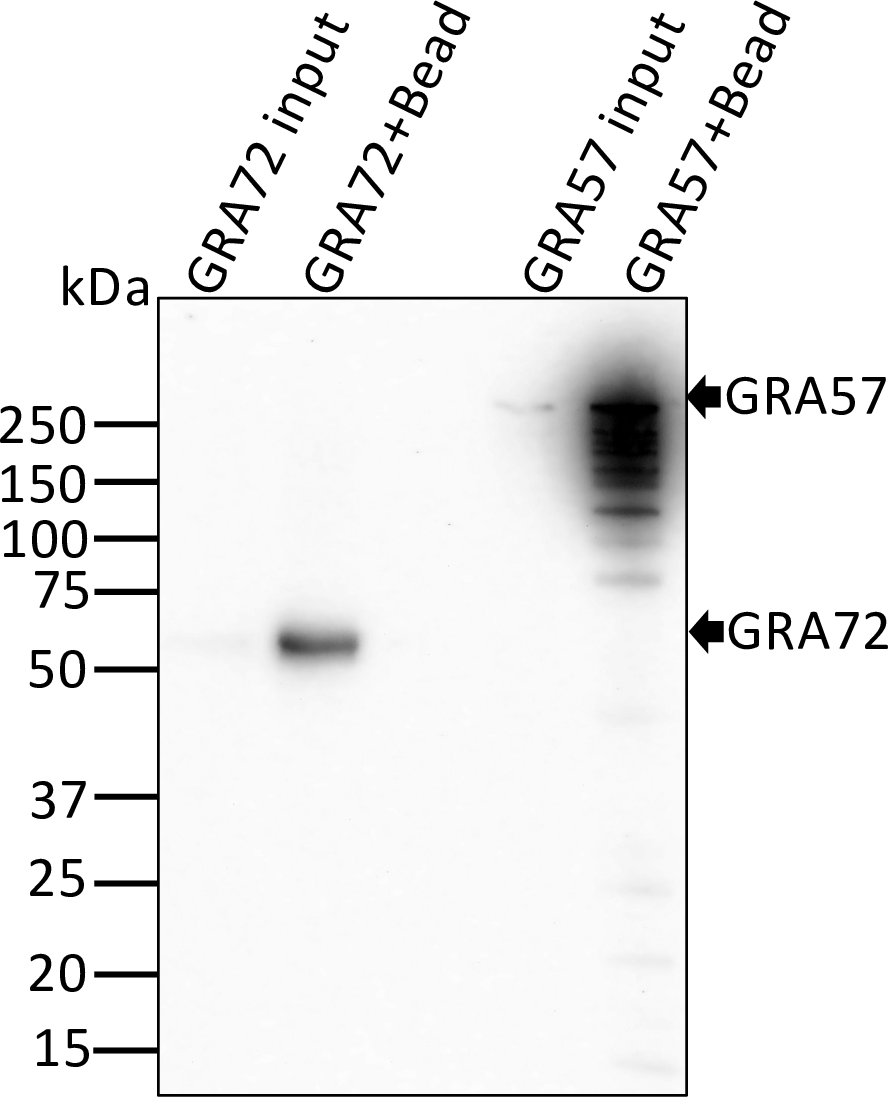
**

**FIG S4** Pulldown of C-terminally HA-tagged GRA72 and GRA57. The Western blot, probed with an anti-HA antibody, displays the successful pulldown of C-terminally HA-tagged GRA72 and GRA57 from the type 1 (RH) background using anti-HA magnetic beads. Predicted molecular weight for GRA57 =246.19kDa and GRA72= 57.51kDa.

**
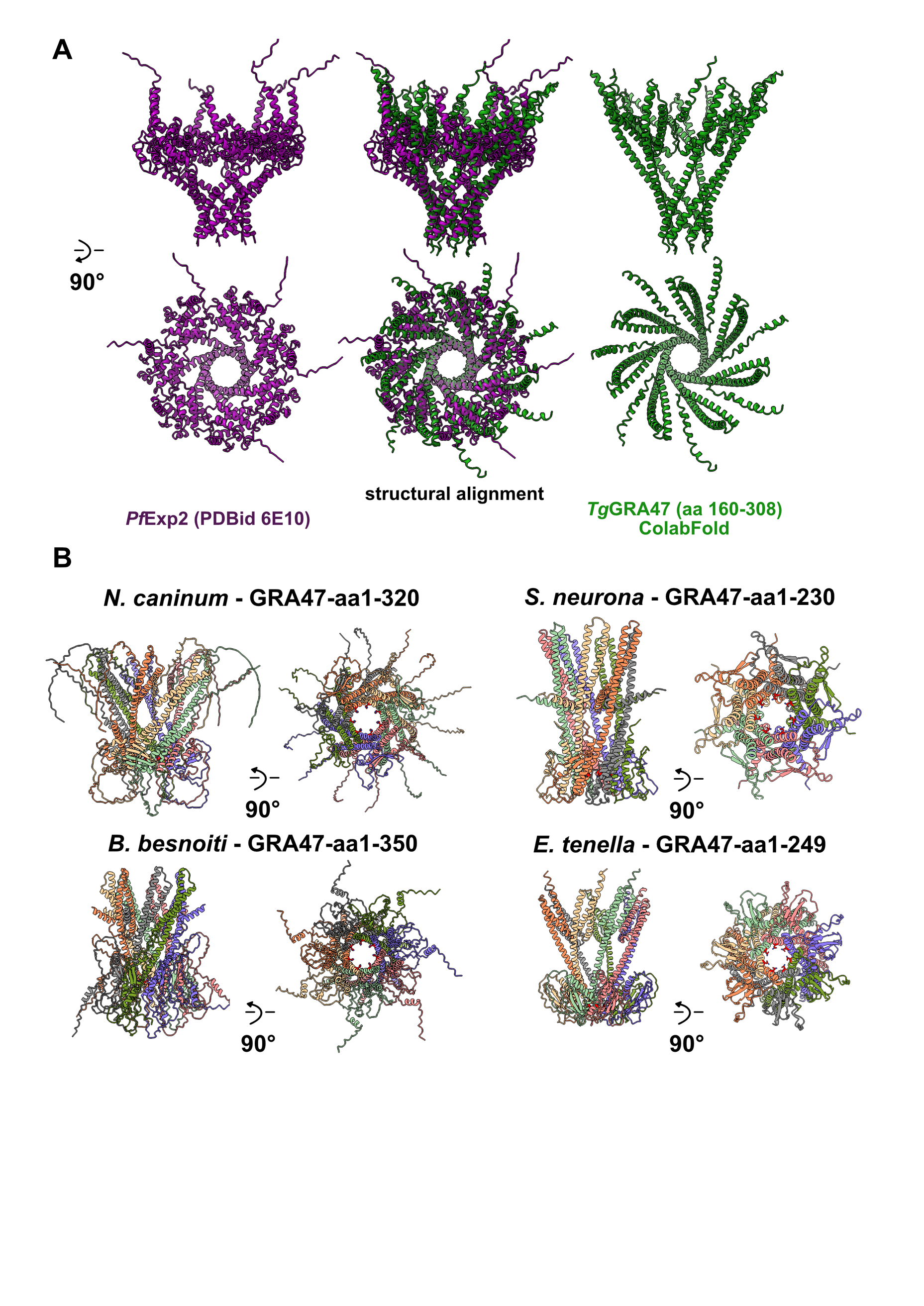
**

**FIG S5** GRA47 orthologs are also predicted to form 7-mer pores**.** **A)** Comparison between the AlphaFold prediction of *Tg*GRA47 and the Exp2 7-mer pore cryo-EM structure. *Pf*Exp2 in magenta and the *Tg*GRA47 (aa 160 to 308) 7-mer model in green were structurally aligned on the N-terminal base of the transmembrane helix and displayed in a cartoon fashion. **B)** Pore forming orthologs of GRA47. Using the same approach as for *Tg*GRA47, *Neospora caninum*, *Sarcocystis neurona*, *Besnoitia besnoiti* and *Eimeria tenella* GRA47 orthologs determined by BLASTp search are also predicted to form 7-mer pore-like structures in AlphaFold-multimer predictions. Rank 1 models are displayed in a cartoon fashion with each monomer colored differently. The 90 ° rotation displays the pore entry with the gating residue at the constriction point shown in red.


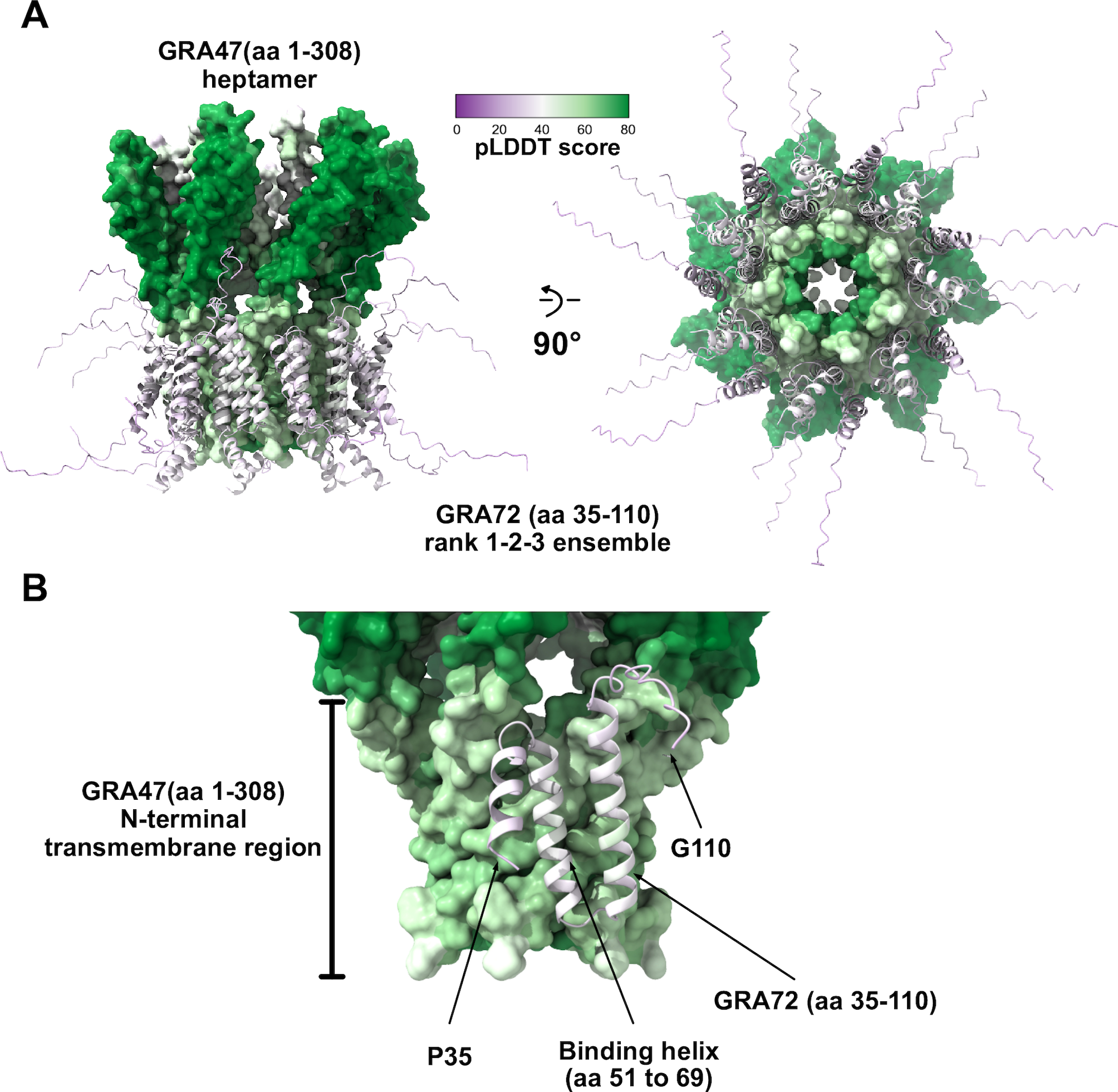


**FIG S6** GRA47/72 7-mer/7-mer heterocomplex prediction. **A)** General view of the GRA47 (aa1-308) 7-mer pore shown in a surface representation with GRA72 (aa35-110) shown as an ensemble of 3 superposed models in cartoon depictions (rank 1 to 3). Colors depict the local pLDDT score on both the surface and cartoon representations. **B)** Focus on GRA72. Using the same rules of representation as panel A, a single model of GRA72 (aa35-110) is shown with the central helix being the main point of contact in this model against the N-terminal region of GRA47.


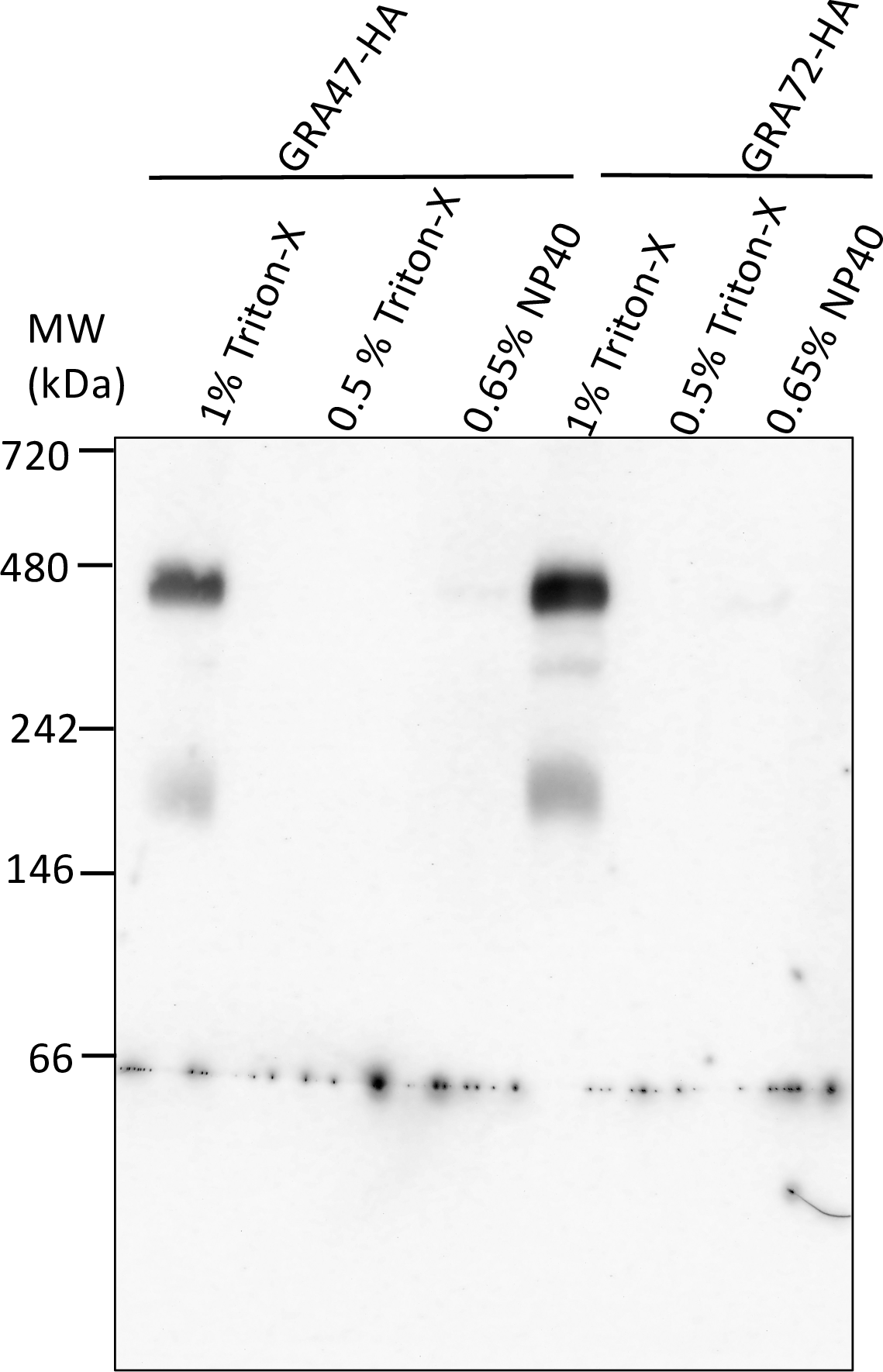


**FIG S7** BluE-Native Page Electrophoresis of GRA47 and GRA72. A pellet collected from HFFs infected with the specified parasites prior to lysis underwent extraction with varying concentrations of detergents. Subsequently, the resultant samples were centrifuged, and the supernatants were loaded for blue native polyacrylamide gel electrophoresis (BN-PAGE). Following electrophoresis, Western blotting was conducted using an anti-HA antibody.


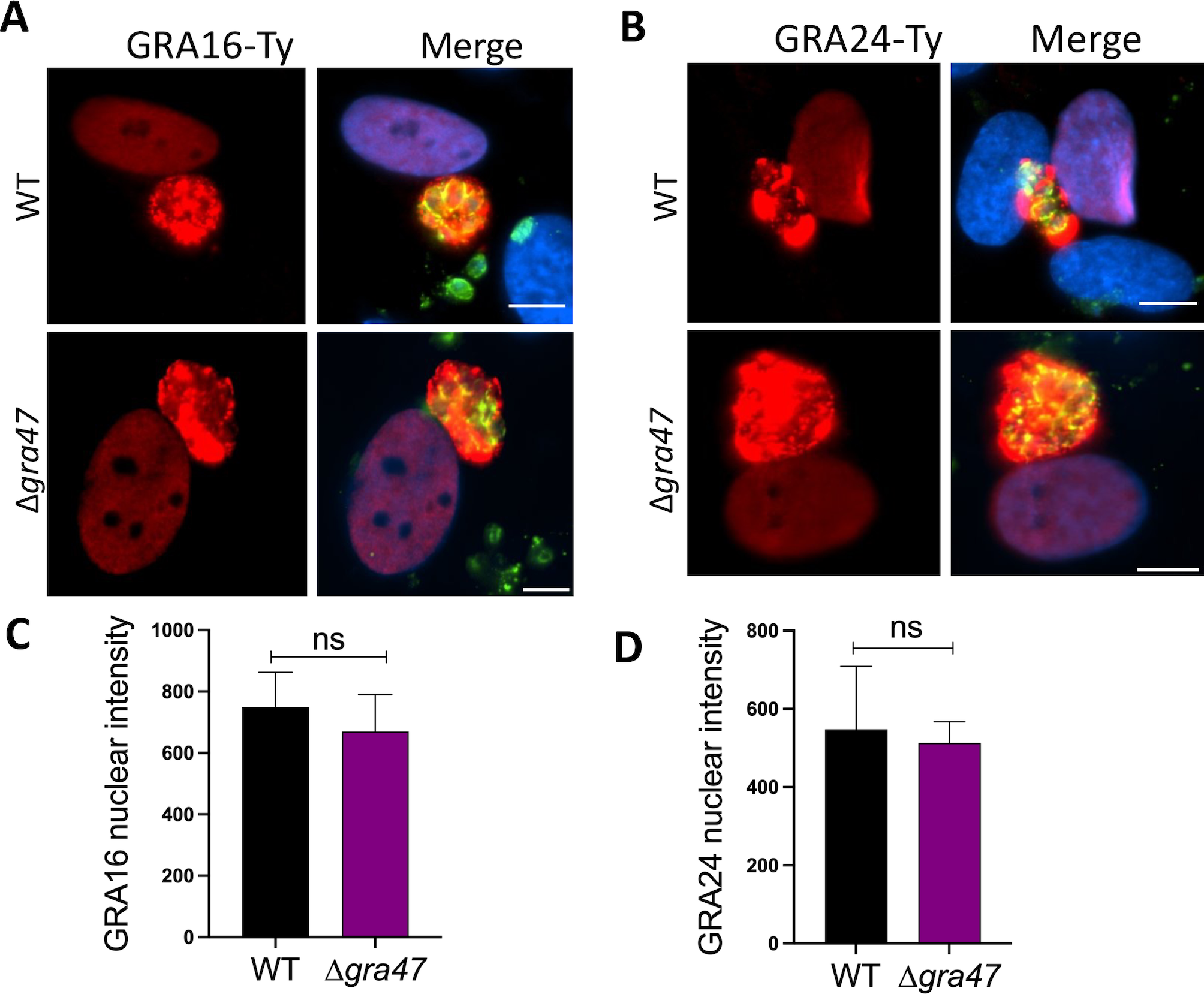


**FIG S8** GRA47 does not affect export of GRA16 or GRA24 to the host cell. HFFs were infected with either wild-type (WT) or Δ*gra47* knockout parasites transiently expressing GRA16-Ty or GRA24-Ty. 24 hours post-infection, the cells were fixed with 3% formaldehyde and subsequently stained using a mouse anti-Ty antibody, shown in red. Panels **A** and **B** display representative images of GRA16 and GRA24 proteins being exported into the host nucleus, respectively. Panels **C** and **D** are the quantification of nuclear signal intensity for GRA16 and GRA24, respectively. Statistical analysis was conducted using a paired T-test (n=3). The images provided are representative of results obtained from three separate experiments, and the scale bars in the images indicate a length of 20 μm. ns= not significant.
